# Role of seed size, phenology, oogenesis and host distribution in the specificity and genetic structure of seed weevils (*Curculio* spp.) in mixed forests

**DOI:** 10.1111/1749-4877.12293

**Published:** 2018-05-17

**Authors:** Harold ARIAS‐LECLAIRE, Raúl BONAL, Daniel GARCÍA‐LÓPEZ, Josep Maria ESPELTA

**Affiliations:** ^1^ CREAF, Centre for Ecological Research and Forestry Applications, Cerdanyola del Vallès 08193 Spain; ^2^ Exact and Natural Sciences School State Distance University of Costa Rica, UNED, Mercedes de Montes de Oca San José Costa Rica; ^3^ Forest Research Group, INDEHESA University of Extremadura Plasencia Spain; ^4^ DITEG Research Group University of Castilla‐La Mancha Toledo Spain; ^5^ Zoology Department Autonomous University of Barcelona, Cerdanyola del Vallès Spain

**Keywords:** *Corylus avellana*, *Curculio* spp., genetic structure, *Quercus* spp., trophic breadth

## Abstract

Synchrony between seed growth and oogenesis is suggested to largely shape trophic breadth of seed‐feeding insects and ultimately to contribute to their co‐existence by means of resource partitioning or in the time when infestation occurs. Here we investigated: (i) the role of seed phenology and sexual maturation of females in the host specificity of seed‐feeding weevils (*Curculio* spp.) predating in hazel and oak mixed forests; and (ii) the consequences that trophic breadth and host distribution have in the genetic structure of the weevil populations. DNA analyses were used to establish unequivocally host specificity and to determine the population genetic structure. We identified 4 species with different specificity, namely *Curculio nucum* females matured earlier and infested a unique host (hazelnuts, *Corylus avellana*) while 3 species (*Curculio venosus, Curculio glandium* and *Curculio elephas*) predated upon the acorns of the 2 oaks (*Quercus ilex* and *Quercus pubescens*). The high specificity of *C. nucum* coupled with a more discontinuous distribution of hazel trees resulted in a significant genetic structure among sites. In addition, the presence of an excess of local rare haplotypes indicated that *C. nucum* populations went through genetic expansion after recent bottlenecks. Conversely, these effects were not observed in the more generalist *Curculio glandium* predating upon oaks. Ultimately, co‐existence of weevil species in this multi‐host‐parasite system is influenced by both resource and time partitioning. To what extent the restriction in gene flow among *C. nucum* populations may have negative consequences for their persistence in a time of increasing disturbances (e.g. drought in Mediterranean areas) deserves further research.

### INTRODUCTION

Seed predation by insects may play a crucial role in plant population dynamics, by reducing the reproductive output (Bonal *et al.* 2007; Espelta *et al.* 2008) and constraining the regeneration process (Espelta *et al.* 2009b). Trophic breadth and specificity of seed-feeding insects is often explained by differences among plant hosts in chemical or morphological traits (Bernays & Graham 1988; Forister *et al.* 2015). Differences in phytochemistry (mainly nitrogen-based defensive compounds) have been observed to be highly relevant for the diversification of phytophagous insects and their diet breadth (Kergoat *et al.* 2005). Concerning other seed features, size is a trait claimed to influence ecomorphological diversification in many endophytic insects (e.g. body size and rostrum shape), promoting differences in their trophic niche, ecological adaptations and species radiation (Hughes & Voegler 2004a; Bonal *et al.* 2011; Peguero *et al.* 2017). In addition to chemical and morphological differences, seeding phenology and stochasticity in the availability of this resource have also been suggested as key factors influencing the guild of insect species predating upon a particular plant host (Espelta *et al.* 2008, 2009b; Coyle *et al.* 2012; see also Pélisson *et al.* 2013a). As insects are short-living organisms, synchronization of their life-cycle with the resources upon which they depend is critical (Bale *et al.* 2002, 2007; Hood & Ott 2010). Therefore, processes such as adult emergence (Espelta *et al.* 2017) and oogenesis (Trudel *et al.* 2002; Son & Lewis 2005) have to be tightly connected with the presence of seeds for oviposition (Bonal *et al.* 2010). In particular, oogenesis (i.e. egg maturation in females) has been predicted to differ depending on the stochasticity of seeds availability. Thus, proovigenesis (i.e. females have already mature eggs at the onset of their adult life) would be favored in species predating upon hosts that regularly produce seeds while synovigenesis (i.e. females start their adult life with immature eggs) would be advantageous for species exposed to more random fluctuations of seed production (Jervis *et al.* 2008; Richard & Casas 2009), as they can better adjust the amount of energy invested in reproduction to the amount of seeds (but see Pélisson 2013b). Ultimately, the co-existence of the different seed consumers in a multi-host community could be mediated by resource partitioning (e.g. insects predate preferentially upon different species according to different seed traits; see Espelta *et al.* 2009a), time-partitioning (e.g. insects exhibit differences in life span and the timing of seed predation; see Pélisson *et al.* 2012) or the trade-off among dispersal versus dormancy ability to cope with resource scarcity (Pélisson *et al.* 2012). Yet, the importance of the interplay among seed size, seeding phenology and oogenesis in driving the guild of insects predating upon seeds of different hosts in multi-specific systems has been seldom explored.

The breadth of the trophic niche of seed-feeding insects (specialist *vs* generalist) may influence the number of species that predate upon different seeds and it has consequences for the dynamics of the community of hosts (Espelta *et al.* 2009b). However, beyond the effects on plant fitness, differences in the trophic niche may also influence the population dynamic of the seed consumers (Ylioja *et al.* 1999) depending on life-history traits such as dispersal ability and landscape attributes (i.e. abundance and spatial distribution of hosts). Spatial connectivity among plant-hosts has been shown to be especially relevant for insects with low dispersal ability (Thomas *et al.* 2001; Kruess 2003), resulting in a stronger genetic structure and reduced gene flow in the insect populations located on more isolated hosts. In the long run, host isolation may even result in colonization credits for some insect species, especially those with a narrower diet breadth (Ruiz-Carbayo *et al.* 2016) and poor dispersal ability (Pélisson *et al.* 2013b; Heineger *et al.* 2014). Conversely, generalist species may show a more continuous distribution in the landscape, benefiting from the spatial overlap of the different host plants upon which they feed (Newman & Pilson 1997), and show no genetic structure differences among populations owing to gene flow. Interestingly, for seed-feeding insects a comparison of the genetic structure of their populations and the spatial structure of their potential hosts could provide strong evidence about differences of the trophic niche breadths. Moreover, the use of molecular techniques (DNA barcoding) may help to detect cryptic speciation and trophic niche segregation among morphologically similar species (Peguero *et al.* 2017), and also to establish species specificity in an unequivocal way in comparison to classifications based on the presence or absence of a species on a particular plant, especially when the lack of morphological differences at certain stages (e.g. larvae) make species identification impossible otherwise (Govindan *et al.* 2012). Unfortunately, this combination of landscape ecology (i.e. host connectivity) and population genetics when studying the breadth of the trophic niche and dispersal ability of phytophagous insects remains largely unexplored.

The main aims of this study have been to investigate in a multi-host and multi-seed-predator system the role of seed size, seed phenology and ooegenesis in the host specificity of seed-parasite weevils (*Curculio* spp.) and to analyze the consequences that potential differences in trophic specialization and host distribution may have in the genetic structure of weevil populations. *Curculio* spp. (Coleoptera: Curculionidae) are seed parasites that differ in their dispersal ability (Venner *et al.* 2011), diapause duration (Pélisson *et al.* 2013a,b), oogenesis (with both proovigenic and synovigenic species; Pélisson *et al.* 2013a) and the breadth of their trophic niche (Muñoz *et al.* 2014; Bonal *et al.* 2015; Peguero *et al.* 2017). We conducted this study in Catalonia (northeast Spain) in mixed forests including oaks (*Quercus ilex*, *Quercus pubescens*) and common hazel trees (*Corylus avellana*) with 4 different weevil species present (*Curculio nucum* Linnaeus, *Curculio glandium* Marsham, *Curculio venosus* Gravenhorst and *Curculio elephas* Gyllenhaal). Interestingly, in this region oaks show a much more continuous distribution and later seeding, while hazels often appear in more discontinuous patches and have an earlier production of fruits (Gracia *et al.* 2004). Concerning weevils, the 4 species overwinter underground, but they differ in the duration of their diapause, the phenology of emergence, oogenesis and dispersal ability. Adults of *C. glandium*, *C. venosus* and *C. nucum* emerge in spring 2 years after larvae buried into the soil, while *C. elephas* exhibits variable diapause and adults emerge in early autumn (Bonal *et al.* 2010; Espelta *et al.* 2017) for up to 3 years (Pelisson *et al.* 2013b). Concerning oogenesis, in *C. glandium*, *C. venosus* and *C. nucum* females are reproductively immature (synovigenic) and ovarian development is accomplished after 1 or 2 months of the feeding period (Bel-Venner *et al.* 2009), while *C. elephas* females are proovigenic and food intake is not required for ovarian development (Pélisson *et al.* 2012). Regarding host selection, previous studies have suggested that *C. nucum* is highly specialized in hazelnuts (Bel-Venner *et al.* 2009), while the other weevils depredate upon several oak species (Muñoz *et al.* 2014). However, these results have not been confirmed by means of DNA analyses as no study has been conducted in mixed hazel–oak forests. Considering the traits of the species involved in this multi-host and multi-predator system and the spatial distribution of hosts, we hypothesize that: (i) seed size and the synchronization of seeding phenology and oogenesis will be responsible for the guild of weevils predating upon the different plants; and (ii) the narrower trophic breadth of *C. nucum* and the more patchy distribution of hazels in comparison to the more generalist habit of the other weevils and the continuous distribution of oaks will result in differences in the genetic structure of weevil populations of these species.

### MATERIALS AND METHODS

#### Study area and species

The study was carried out in mixed forests with the presence of oaks (*Q. ilex* and *Q. pubescens* and common hazel trees (*Co. avellana*) in Catalonia (north-east Spain, Fig. 1). The evergreen *Q. ilex* and the winter-deciduous *Q. pubescens* are extensively distributed in pure and mixed forests in all the western rim of the Mediterranean basin (Espelta *et al.* 2008), while the common hazel (*Co. avellana*) often appears in scattered groups in mixed deciduous forests or cultivated in monospecific stands (AliNiazee 1998). Acorns in *Quercus* spp. and hazelnuts in *Co. avellana* mature in 1 year and both are subjected to intense pre-dispersal seed predation by weevils (*Curculio* spp.), a group of granivorous insects extensively distributed in the northern hemisphere (Hughes & Voegler 2004a). In Catalonia, the most common weevil species predating upon acorns are *C. glandium* and *C. elephas* (Espelta *et al.* 2009b), the latter also depredating upon chestnuts (*Castanea* spp.), while in hazelnuts the unique species described up to now has been *C. nucum*, a hypothesized highly specific seed parasite (Guidone *et al.* 2007; Bel-Venner *et al.*

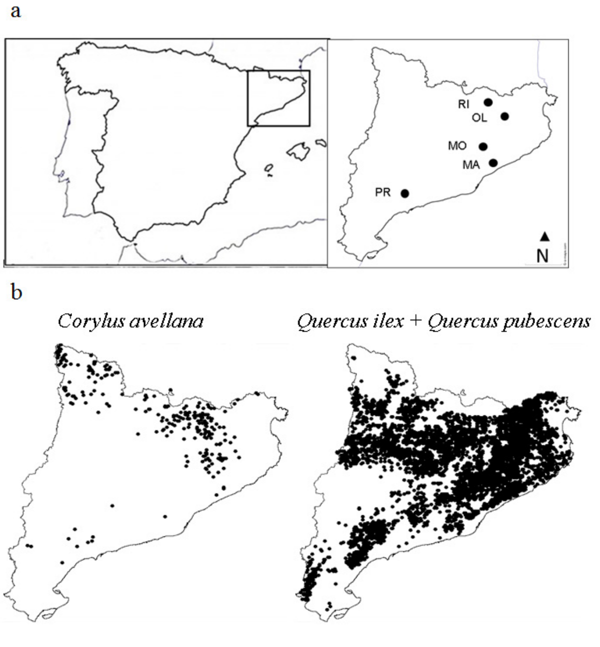

**Figure 1** (a) Location of study sites in Catalonia (north-east Spain). (b) Distribution of *Corylus avellana*, *Quercus ilex* and *Quercus pubescens* according to the presence of this species in plots inventoried in the Catalan Forest Inventory (Gracia *et al.* 2004). RI, Ripoll (5 plots); OL, Olot (4 plots); MO, Montseny (4 plots); MA, Maresme (5 plots); PR, Prades (5 plots).

2009). However, it must be highlighted that except for the weevil species predating upon oaks, ascription of weevil species to a plant host is based on the observation of adults in the foliage of that particular species, but no study has addressed this issue comprehensively (e.g. identifying by means of molecular techniques the species of the larvae inside chestnuts or hazelnuts).

During early summer on hazelnuts (AliNiazee 1998) and early autumn on acorns (see Bonal & Muñoz 2009) female weevils perforate the seed cover with their snout and oviposit commonly a single egg so the larvae develop feeding on the seed kernel. At the middle of summer in *C. nucum* (Bel-Venner *et al.* 2009) or late autumn in *C. elephas* and *C. glandium* (Espelta *et al.* 2009a), larvae exit the seed and bury into the ground to overcome the diapause period and undergo full metamorphosis.

### Sampling design

In 2013 we established a total of 23 sampling plots grouped into 5 geographical clusters (Sites) in a north to south latitudinal gradient (see Fig. 1). This sampling procedure was selected to account for the possible effects of latitude on the duration of the vegetative season and, thus, on the seeding phenology of oaks and hazelnuts, their overlap and the overlap among these host species and the weevils predating upon their seeds. Presumably a tighter vegetative season in northern and colder sites would lead to more similar patterns of seed production while these could be more relaxed and longer in southern and warmer places. Ultimately, this could lead to differences in the guild of weevils predating upon these plants. Plots were selected by searching for the presence of trees of *Co. avellana* and *Q. ilex* or *Q. pubescens* based on the Catalan Forest Inventory (Gracia *et al.* 2004) and field observations of their reproductive status (i.e. presence of seeds). From late July (end of hazel seeding season) to early October (end of the acorn crop) we carried out 3 sampling campaigns: (i) late July to early August; (ii) late August to early September; and (iii) late September to early October to account for possible differences in the phenology of seed infestation by the different weevil species present. In every plot and in each sampling period we randomly collected a minimum of 100 seeds from each species (*Co. avellana* and *Quercus* spp.) under the canopies of several randomly selected trees. Seeds were taken to the laboratory and classified as sound or infested to assess infestation rates per species and sampling period. Infested seeds are easily recognizable by the presence of female oviposition scars. We calculated the volume of both sound and infested seeds by measuring the length and width to the nearest 0.01 mm with a digital caliper (see Espelta *et al.* 2009a). Infested seeds were placed individually in plastic trays for individual monitoring. Each seed was checked daily to register the emergence of larvae, which were immediately transferred to 2 mL Eppendorf with 96% alcohol. Once larvae stop emerging (approximately 3 weeks after seeds were collected) seeds were dissected to check for the presence of non-emerged larvae. From hazelnuts only *Curculio* larvae emerged, while for acorns the 6% of larvae corresponded to the *Cydia* spp. moth.

In parallel, during the abovementioned field campaigns adult weevil were captured by shaking the canopy and collecting the fallen individuals in an inverted umbrella held beneath the foliage for species identification at the laboratory. To establish whether females were sexually mature, they were dissected under a microscope to observe abdominal segments and ovary maturity. We considered the presence of eggs as a sign of female ready for oviposition and the absence of eggs as females that were still immature or had already oviposited (Pélisson *et al.* 2013a).

### DNA barcoding and larval species identification

A total of 1657 *Curculio* larvae emerged from hazelnuts and acorns. In order to establish unequivocal trophic relationships between insects and their host plants we used molecular techniques (DNA barcoding) as larvae cannot be determined according to morphological characters. Therefore, from 342 larvae selected randomly among the ones emerged in the laboratory from the 3 hosts we extracted DNA from a small piece of larval tissue (approximately 2-mm long) using the NucleoSpin-Tissue kit according to the manufacturer's instructions (MACHEREY-NAGEL GmbH, Düren, Germany; www.mn-net.com). We amplified a fragment (826 bp) of the mitochondrial cytochrome oxidase subunit 1 (cox1) using primers Pat and Jerry (please see Hughes & Vogler [2004b] for details on primer sequences and PCR protocols). We chose this fragment of cox1 due to the availability of many reference sequences from correctly determined adults of European *Curculio* spp. For comparison (Hughes & Vogler 2004b). Sequencing was performed using Big-Dye (Perkin-Elmer) technology and an ABI3700 sequencer. Sequence chromatograms were assembled and edited using Sequencher 4.6 (Gene Codes, Ann Arbor, MI, USA). For species identification we discarded those sequences that after edition were shorter than 500 base pairs. Edited sequences were aligned using CLUSTALW supplied via http://align.genome.jp, with default gap open and gap extension penalties. The alignment sets were collapsed into unique haplotypes and each of this compared to the *Curculio* spp. reference sequences available at GenBank. We applied the most conservative limit used in DNA barcoding, which states a maximum genetic divergence (number of different nucleotides by the total number of compared nucleotides) of 1% with respect to the reference sequence for an unambiguous identification to the species level (Ratnasingham & Herbert 2007).

#### Data analysis

To evaluate the occurrence of the different weevil species in the 3 potential hosts *Co. avellana*, *Q. ilex* and Q. *pubescens*) across the 5 study sites, we conducted a Pearson's $\chi^2$-test. Similarly, we used $\chi^2$-tests for the comparison of the presence of male and female weevil proportion, as well as that of immature and mature females among sampling periods. A generalized linear mixed model (GLMM), following a binomial distribution, was used to test for the effects of the study site (RI, Ripoll; OL, Olot; MO, Montseny; MA, Maresme; PR, Prades), sampling period (1, 2, 3) and host species (*Co. avellana*, *Q. ilex* and *Q. pubescens*) on the seed predation rate by weevils. The factor "plot" was included as a random effect in the GLMM analyses to account for the repeated nature of the measurements and other unexplained variation. Analyses of deviance Type II Wald $\chi^2$-tests were performed to establish the significance of each different independent variable in the model. A general linear mixed model was applied to test for the effects of host species, sampling period and seed condition (sound or infested) on seed size (volume in $mm^3$) with the factor "plot" included as a random effect.

For population genetic analyses we chose those species in which there were a minimum of 10 individuals per population with sequences longer than 750 bp; namely, *Curculio glandium* and *Curculio nucum*. We used ARLEQUIN software (Excoffier *et al.* 2005) to calculate standard molecular diversity indices (gene diversity and nucleotide diversity) and to perform analyses of the molecular variance (AMOVAs). Signatures of population demographic changes (bottlenecks or expansions) were examined by Tajima's *D* (Tajima 1989) and Fu's *F* (Fu 1997) as implemented in ARLEQUIN software. We also tested whether there was any geographic pattern in the population genetic structure using SAMOVA 1.0 (Dupanloup *et al.* 2002). This method identifies the optimal grouping option (K) that maximizes the among-group component (FCT) of the overall genetic variance. We defined the number of populations (K) and ran 100 simulated annealing processes. We simulated different numbers of populations, ranging from $K = 2$ to $K = 4$, to determine the best population clustering option.

### RESULTS

Molecular analyses allowed the identification of the larvae emerged from the seeds of the 3 host species (*Co. avellana*, *Q. ilex* and *Q. pubescens*) as all sequences showed a divergence below 1% with respect to *Curculio* spp. reference sequences from GenBank. This divergence was much lower than inter-specific differences, which in all cases exceeded 8%. All larvae correspond-ed to 4 species; namely, *C. elephas*, *C. glandium*, *C. nucum* and *C. venosus*. As shown in Figure 2, weevil species were not randomly distributed among hosts; that is, *C. nucum* was exclusively present in hazelnuts while the other 3 weevils emerged uniquely from acorns ($\chi^2_6 = 263.9$, $P < 0.001$). *C. glandium* and *C. elephas* were more abundant in *Q. ilex* (respectively, $\chi^2_{14} = 91.8$, $P < 0.001$, and $\chi^2_8 = 23.3$, $P < 0.001$) while there were not significant differences in the presence of *Curculio venosus* between the 2 oak species ($\chi^2_5 = 7.47$, $P > 0.05$). The different presence of larvae of the 4 weevil species

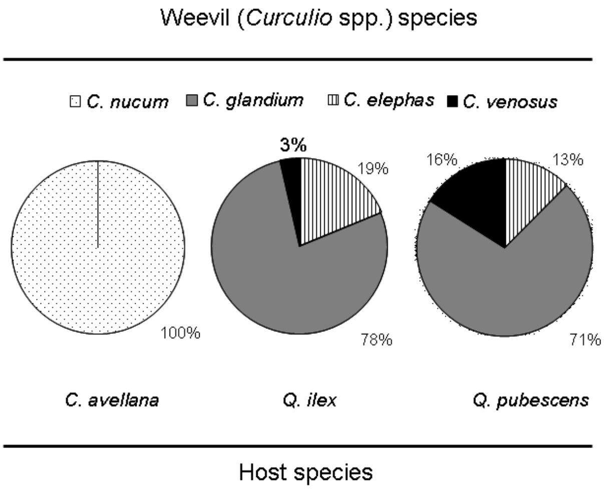

**Figure 2** Proportion of the different weevil species infesting the seeds of the 3 host species (*Corylus avellana*, *Quercus ilex* and *Quercus pubescens*) according to the DNA analyses of the larvae emerging from the seeds.

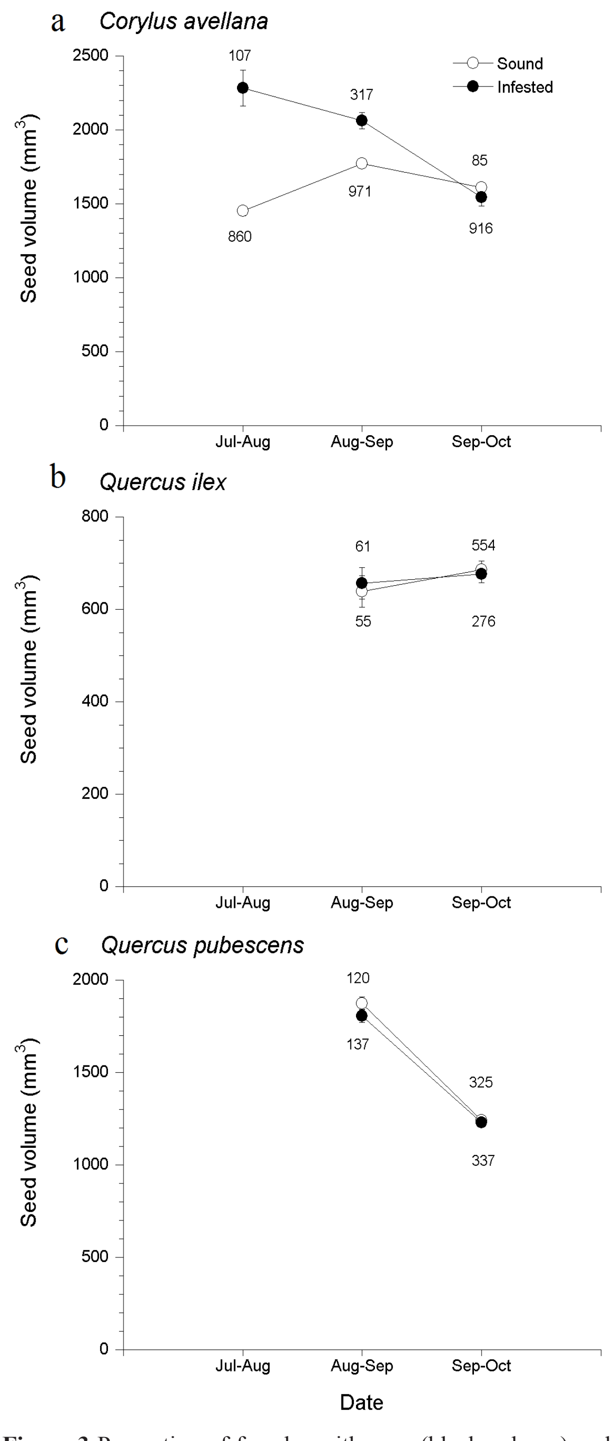

**Figure 3** Proportion of females with eggs (black column) and without eggs (white columns) for the 3 weevil species (*Corylus avellana*, *Quercus ilex* and *Quercus pubescens*) captured in the 3 sampling periods during the seeding season: Jul–Aug = from the end of July to the beginning of August; Aug–Sep = from the end of August to the beginning of September; Sep–Oct = from the end of September to the beginning of October.

in the 3 hosts, especially among hazelnut and the 2 oaks, was not due to the lack of a particular species in a given site as we captured adult specimens of all weevil species along the entire gradient. Moreover, as hazelnuts were bigger than acorns during almost the entire seeding season (Table 1 and Fig. 3), the abovementioned differences in weevil specificity among these 2 groups of hosts does not seem to be due to the exclusion of certain weevil species from hazelnuts by a too small seed size.

Male and female weevils occurred in nearly the same frequency with no significant variation along the sampling periods ($\chi^2_2 = 2.28$, $P > 0.05$). Yet the proportion of females with presence of eggs and without eggs showed significant differences through the season ($\chi^2_2 = 33.7$, $P < 0.001$) and for the different weevil species. As shown in Figure 4, through the season the presence of females with eggs was earlier in *C. nucum*, followed by *C. glandium* and *C. elephas*. In the 2 synovigenic species, *C. nucum* had a decreasing pattern ($\chi^2_2 = 10.5$, $P < 0.01$) in the presence of females with eggs, while *C. glandium* exhibited an increasing pattern ($X^2_2 = 35.3$, $P < 0.001$). In the proovigenic *C. elephas* we did not find females on the very first sampling period but as soon as they appeared during the second and third sampling dates they were already sexually mature ($\chi^2_2 = 16.5$, $P < 0.001$). Consistently with the seasonal patterns of the presence of females ready to oviposit, we found that infestation rates showed significant variation among study sites ($\chi^2_4 = 16.5$, $P < 0.001$), sampling periods ($\chi^2_1 = 5.5$, $P < 0.05$) and host species ($\chi^2_2 = 6.4$, $P < 0.05$). Overall, infestation was higher in northern localities and it increased as the seeding season progressed (see coefficients for the different effects in Table 2). Concerning host species, infestation rates showed contrasting temporal patterns in hazelnut versus oaks (Table 2, Fig. 5), in agreement with host seeding phenology and oogene-

**Table 1** Estimates for the significant effects of tree host, sampling period and seed condition (sound or infested) over seed size (volume in mm$^3$) according to the linear mixed model

| Effects | Estimate | Standard error | *t*-value |
|---|---|---|---|
| Intercept | 3.199 | 0.0278 | 153.912*** |
| Date | 0.01695 | 0.004249 | 3.990*** |
| Host, *Q. pubescens* | −0.1876 | 0.008793 | −21.336*** |
| Host, *Q. ilex* | −0.4808 | 0.009984 | −48.159*** |
| Seed condition, sound | −0.03143 | 0.006962 | −4.515*** |

*$P < 0.05$, **$P < 0.01$ and ***$P < 0.001$.

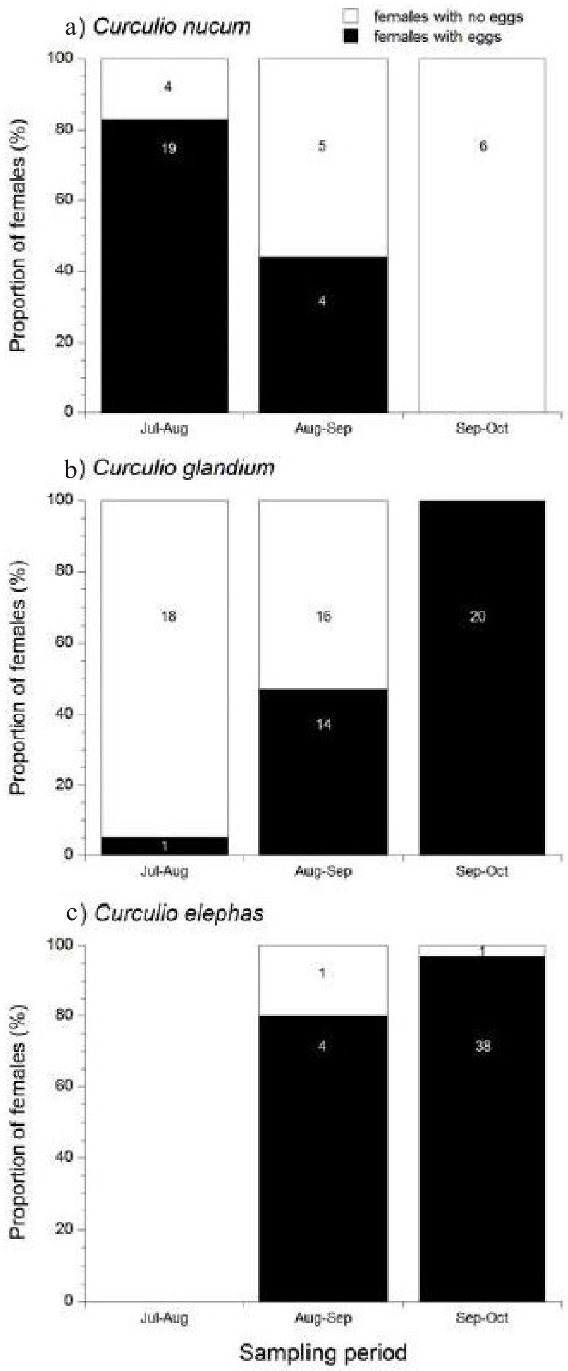

**Figure 4** Mean ± SE volume ($mm^3$) of sound (open dots) and infested (black dots) seeds of the 3 host species (*Corylus avellana*, *Quercus ilex* and *Quercus pubescens*) along the sampling dates. Jul–Aug = from the end of July to the beginning of August; Aug–Sep = from the end of August to the beginning of September; Sep–Oct = from the end of September to the beginning of October. Notice the difference in the scale of the *y*-axis for the 3 host species.

**Table 2** Estimates for the significant effects of study site, tree host and sampling period on weevil infestation rates according to the generalized linear mixed model

| Effects | Estimate | Standard error | *z*-value |
|---|---|---|---|
| Intercept | −2.8280 | 0.5462 | −5.177*** |
| Site Olot | 1.0830 | 0.4776 | 2.268* |
| Site Ripoll | 1.5611 | 0.4788 | 3.260** |
| Host, *Q. pubescens* | 0.9615 | 0.3810 | 2.523* |
| Date | 0.4634 | 0.1973 | 2.349* |

*$P < 0.05$, **$P < 0.01$ and ***$P < 0.001$.

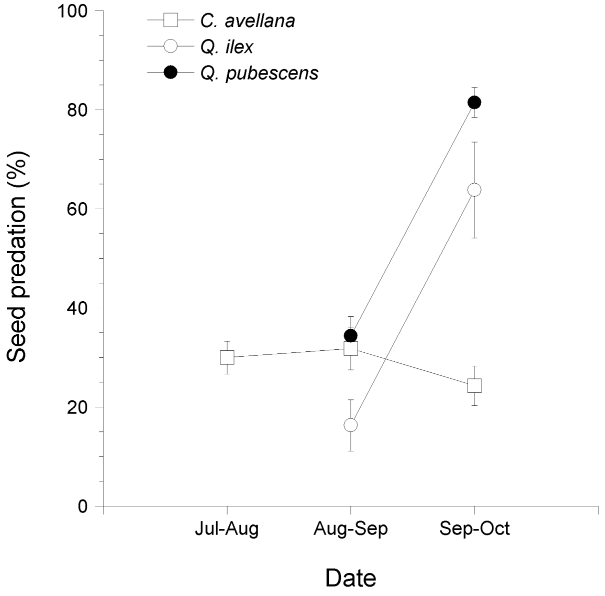

**Figure 5** Mean ± SE seed infestation rate of the 3 host species (*Corylus avellana*, *Quercus ilex* and *Quercus pubescens*) along the sampling dates. Jul–Aug = from the end of July to the beginning of August; Aug–Sep= from the end of August to the beginning of September; Sep–Oct = from the end of September to the beginning of October.

sis in females; that is, in hazelnuts infestation occurred earlier and slightly decreased through the season, while it was absent during the first sampling date in the 2 oak species, and progressively increased towards the end of the season (Fig. 5).

The population genetic analyses showed marked differences between *C. nucum* and *C. glandium*. Mean genetic diversity was higher in *C. nucum* (Table 3), mainly due to the higher number of distinct haplotypes; that is, an ANOVA in which the population was included as

**Table 3** Values of gene diversity, nucleotide diversity, Tajima's *D* and Fu's *F* recorded at each population for *Curculio nucum* (a) and *Curculio glandium* (b)

| | Gene diversity | Nucleotide diversity | Tajima's *D* | Fu's *F* |
|---|---|---|---|---|
| (a) *Curculio nucum* | | | | |
| Ripoll | 0.87 | 0.0015 | −1.63* | −4.54*** |
| Olot | 0.75 | 0.0024 | −1.96** | −8.34*** |
| Montseny | 0.89 | 0.0032 | −1.40* | −6.87*** |
| Maresme | 0.88 | 0.0034 | −0.41 | −1.20 |
| Prades | 0.59 | 0.0012 | −1.69** | −5.27*** |
| (b) *Curculio glandium* | | | | |
| Ripoll | 0.71 | 0.0012 | −1.10 | −2.61* |
| Olot | 0.57 | 0.0009 | −0.46 | −0.84 |
| Montseny | 0.69 | 0.0013 | −1.22 | −2.61* |
| Maresme | 0.69 | 0.0013 | −0.75 | −1.95 |
| Prades | 0.63 | 0.0034 | −1.79 | 1.46 |

*$P < 0.05$, **$P < 0.01$ and ***$P < 0.001$.

a random factor showed that mean gene diversity was significantly higher in *C. nucum* ($F_{1,4} = 9.40$; $P = 0.03$). A total of 31 haplotypes were retrieved from the 118 sequences of *C. nucum* included in the analyses versus just 13 from 96 sequences in *C. glandium* (see Tables 4 and 5). In *C. nucum*, 48% of the individuals had the most common haplotype but there were many rare haplotypes sometimes found in just one individual and/or at a single population (Table S1). In the case of *C. glandium*, there were much fewer rare haplotypes and the two most frequent ones were found in 80% of the individuals (Table S2). The high proportion of rare haplotypes in *C. nucum* suggests population expansion after recent bottlenecks and, accordingly, both Tajima's D and Fu's F had negative and significant values in all populations except one. In the case of *C. glandium* only for Fu's test were the values significant in two populations, thus showing that most populations were in equilibrium (Tajima 1989; Fu 1997). The AMOVA revealed a more restricted gene flow between populations in the case of *C. nucum*, in which differentiation among populations explained 5.02% of the total molecular variance (degrees of freedom = 4; $P < 0.01$), whereas in *C. glandium* inter-population differences were not significant. The results of the SAMOVA were marginally significant for *C. nucum* (FCT = 0.08; degrees of freedom = 1; $P = 0.08$) and defined two clusters within the geographical range of our study, the first grouping the nearby populations of Montseny (MO) and Maresme (MA) and another one including the rest (see Fig. 1). No significant geographical pattern of molecular variance was found in *C. glandium*.

## DISCUSSION

Seed infestation by weevils did not occur randomly but with 2 opposite breadths of host specificity; namely, the highly specialized *C. nucum* infested a unique host (hazelnuts), while up to 3 species (*C. glandium*, *C. elephas* and *C. venosus*) predated almost indistinctively upon 2 oaks (*Q. ilex* and *Q. pubescens*). These differences in trophic specificity coupled with differences in the geographical distribution of the hosts resulted in 2 distinct patterns concerning the genetic characteristics of weevils' populations; that is, we only found a significant genetic structure among the populations in the highly specialist *C. nucum*. Ultimately, the results of these genetic analyses confirmed the specialist or generalist trophic breadth of the different weevil species according to the identification of the larvae found in the seeds and they stress how molecular techniques may help to establish unequivocal trophic relations for seed feeding insects.

Previous studies have suggested that seed size has been a relevant trait promoting ecomorphological adaptations in the genus *Curculio* and driving species diversification (Hughes & Vogler 2004a; see also Peguero *et al.* 2017). In the end, a tight relationship between seed and weevils' body size would result in differences in the ability of larger and smaller weevils to infest seeds of different size (differences in trophic breadth); that is, small species would be able to infest both small and large seeds while species with a larger body size would be restricted to larger seeds to obtain enough resources to complete larvae development (see Bonal & Muñoz 2008; Espelta *et al.* 2009a; Bonal & Muñoz 2011; Peguero *et al.* 2017). Yet, this does not seem to be the case in our study system where hazels, the species infested by a single species (*C. nucum*), showed the largest seeds during most of the season (Fig. 3) and experienced the lower infestation rate (see Table S3). Instead of an influence of seed size, our results suggest that the exclusive infestation of hazelnuts by *C. nucum* could be more related to a different pattern of sexual maturation of females among the 2 weevil species emerging from the soil in spring, specifically an earlier maturation in *C. nucum* in comparison to *C. glandium* (Fig. 4). These differences could be due to differences between the 2 weevils in the requirements of resource acquisition as it has been demonstrated that sexual maturation in females of these synovigenic species critically requires some feeding at adulthood before reproductive development takes place (Bel-Venner *et al.* 2009; Pélisson *et al.* 2012). The early maturation in *C. nucum* would be advantageous to oviposit in hazelnuts before the hardening of the nutshell, as this is a fast process occurring during seed growth and the main mechanism in hazels to avoid infestation (Guidone *et al.* 2007). Moreover, oviposition of *C. nucum* would be expected to occur soon after mating as weevils do not adjust laying eggs to the moment of highest seed availability, but they oviposit as soon as females have mature eggs (Bonal *et al.* 2010). This behavior is probably linked to the temporal unpredictability of seed crop size (Bonal & Muñoz 2008; Espelta *et al.* 2008) and other constraints they have to cope with, such as the need of rainfall episodes to soften the soil and allow the emergence of adults (Bonal *et al.* 2010, 2015; Espelta *et al.* 2017). Only during the 2 earlier samplings, females of *C. nucum* seemed to preferentially choose bigger seeds, a behavior related with the need to select a minimum seed size to ensure larvae development and also owing to the availability of more seeds for oviposition (see Espelta *et al.* 2009a).

In comparison to the extreme host–parasite specificity of *C. nucum*, the other 3 weevils (*C. venosus*, *C. glandium* and *C. elephas*) predated indistinctively upon the 2 oaks with no evidence of a strategy in the partitioning of this resource according to the identity of the host species or to seed size. The avoidance of competitive exclusion among these species could be explained by several mechanisms contributing to stabilize their coexistence; that is, time partitioning (Pélisson *et al.* 2012; see also Espelta *et al.* 2009a) and/or diversification of dispersal-dormancy strategies (Pélisson *et al.* 2012). On the one hand, time partitioning in breeding activity can exist when 2 competing species differ in the speed of energy acquisition to be allocated to reproduction by females and the duration of their lifespan; that is, one species acquires resources faster and it is able to oviposit earlier on seeds, but it is exposed to a higher risk of seed abortion, while the other oviposits later but has a longer life span allowing it to lay eggs during a larger time frame (see Pelisson *et al.* 2012 for *C. pellitus* and *C. glandium*). On the other hand, stabilization can be reached by means of different dispersal versus dormancy strategies with some species relying on a high dispersal ability and others depending on dormancy strategies (e.g. variable diapause) to cope with seed scarcity. This seems to be the case for *C. glandium* and *C. elephas*; that is high dispersal ability (up to 11 km) in the former species and an extended diapause (up to 3 years) in the later (see Venner *et al.* 2011; Pélisson *et al.* 2012). Yet, other factors not covered in this study, such as the risk of parasitism or survival of larvae during diapause, may also help equalize their success to infest (Bonal *et al.* 2011). Similarly, future studies with more intense and appropriate sampling schemes should address the relationship between the number of adults of the different species and the number of larvae to disentangle the different predation rates upon each species and the influence of other environmental factors.

Ultimately, differences in the trophic breadth leave a contrasting genetic signature in the populations of the 2 species of weevils. A much higher number of local rare haplotypes were found in the monophagus *C. nucum*, along with a marginally significant genetic structure among populations, contrary to the more generalist *C. glandium* (see, for a similar example in aphids, Gaete-Eastman *et al.* 2004). Inter-specific differences in genetic characteristics of phytophagous insects could arise from differences in their dispersal ability or in the spatial distribution (isolated *vs* continuous) of the host (Peterson & Denno 1998; Kubish *et al.* 2014). Unfortunately, in comparison to the precise information about the dispersal ability of *C. glandium* (approximately 10 km in Pélisson *et al.* 2012), we lack detailed knowledge about the dispersal range of *C. nucum*, except some evidence of weevils moving away from local sites to feed during adulthood and prior to mating (Bel-Venner *et al.* 2009). Yet the fact that *C. nucum* and *C. glandium* are sister species (Hughes & Voegler 2004a) and they share many similar life-history traits, such as ecomorphological adaptations and body size, adult emergence in spring, synovigenic females and a fixed diapause of 2 years (see Hughes & Vogler 2004a; Bel-Venner *et al.* 2009; Pélisson *et al.* 2012a,b), make us consider that they may have a similar dispersal ability. Therefore, the differences we observed in their genetic characteristics would be probably due to their different diet breadth and the more patchy and discontinuous distribution of hazels in comparison to the more abundant and constant presence of oaks (Gracia *et al.* 2004; see also Fig. 1), along the geographical gradient sampled (approximately 225 km from Ripoll to Prades).

Connectivity may be critical for population survival (Fahrig & Merrian 1985; Fahrig & Paloheimo 1988) and metapopulation dynamics (Levins 1970), especially in front of a disturbance: for example, the negative impact of severe drought episodes for the emergence of adult weevils (Bonal *et al.* 2015; Espelta *et al.* 2017). In that sense, our molecular data show that such disturbances may have occurred and left their signature in *C. nucum* population genetics. The significant negative values retrieved in the neutrality tests (Tajima's D and Fu's F) indicate that most of the *C. nucum* populations sampled went through population expansion after recent bottlenecks. Almost half of the individuals had the same haplotype and there was an excess of rare haplotypes that differed little from the most common one. The lower gene flow between populations (marginally significant genetic structure among populations) in *C. nucum* would favor such bottlenecks as the patchy distribution of hazel trees would complicate the arrival of immigrants. None of this happened in the case of *C. glandium* feeding on the widespread oak trees. Yet the interpretation of the results for *C. nucum* must be cautious as the shallow genetic structure observed suggests that a fair amount of gene flow still occurs, enough to overcome drift. Moreover, in addition to the current distribution of hazelnuts, other abiotic environmental conditions could also be involved in the genetic structure observed in *C. nucum* (e.g. geological barriers or altitude for *Trichobaris soror* in De la Mora *et al.* 2015)

### CONCLUSION

The use of molecular analyses allowed us to precisely identify the weevil species depredating upon the various potential hosts in these mixed deciduous forests and to unequivocally confirm the high specificity of the hazelnut *C. nucum* and the more flexible and wider trophic breadth of the rest of the weevils (*C. venosus*, *C. glandium* and *C. elephas*) depredating upon acorns. In this multi-host and multi-parasite system, co-existence of the various weevil species seems to be mediated by a combination of extreme resource partitioning (i.e. among *C. nucum* and the rest of species) and a combination of time partitioning and differences in dispersal-dormancy strategies among the 3 species depredating upon oaks. Interestingly, although sometimes suggested, differences in seed size did not have any effect in driving host specificity or the trophic breadth of the weevil species present. Moreover, our results highlight that differences in specificity in trophic breadth and in the spatial distribution of hosts at a large geographical scale may result in the presence of genetic structure among the populations of highly specific parasites (*C. nucum*) depredating upon patchily distributed hosts (*Co. avellana*). To what extent this restriction in gene flow (dispersal) may have negative consequences for the persistence of the populations of these highly specialized seed-feeding pests in a time of increasing disturbances (e.g. drought events in Mediterranean areas) is a fascinating question that deserves further research.

### ACKNOWLEDGMENTS

This research was supported by the projects FOR-ASSEMBLY (CGL2015-70558-P) and PLAGANADO (AGL2014-54739-R) of the Spanish Ministry of Economy, and the projects BEEMED (SGR913) (Generalitat de Catalunya) and PII1C09-0256-9052 (Regional Government of Castilla-La Mancha and the European Social Fund). R.B. was funded by a contract of the Program Atracción de Talento Investigador (Gobierno de Extremadura).

### SUPPLEMENTARY MATERIALS

**Cite this article as:**

## Cite this article as:

Arias‐LeClaire H, Bonal R, García‐López D, Espelta JM (2018). Role of seed size, phenology, oogenesis and host distribution in the specificity and genetic structure of seed weevils (*Curculio* spp.) in mixed forests. *Integrative Zoology* **13**, 267–79.

## Supporting information

Additional supporting information may be found in the online version of this article.
**Table S1** Number of *Curculio nucum* individuals bearing each haplotype in the 5 study populations
**Table S2** Number of *Curculio glandium* individuals bearing each haplotype in the 5 study populations
**Table S3** Mean ± SE density of host plants and the percentage of sound and infested seeds per location and host plant Density of host plants was calculated as the mean of the nearest inventoried plots included in the Catalan Forest Inventory (Gracia *et al*. 2004).

Supporting Information
