# Supplementary material for: Role of seed size, phenology, oogenesis and host distribution in the specificity and genetic structure of seed weevils (Curculio spp.) in mixed forests
Source: Integr Zool. 2018 May 17;13(3):267–79. doi: 10.1111/1749-4877.12293 (PMC6221125; doi:10.1111/1749-4877.12293)
Supplement: Supplementary file 1 — Supporting Information [file INZ2-13-267-s001.pdf]

Table S1 Number of *Curculio nucum* individuals bearing each haplotype in the five study populations

**Haplotype**	Maresme	Montseny	Olot	Prades	Ripoll
**1**					2
**2**					1
**3**	1	1		1	
**4**		1			
**5**					1
**6**					1
**7**	2	3			
**8**		1		1	
**9**		1			
**10**	1				
**11**	1	7	13	28	4
**12**	3	4		1	
**13**	1	1	1		
**14**		1			
**15**		1	1		
**16**		1			
**17**		1			
**18**			1		2
**19**	1		1		
**20**			1		
**21**		1	2		
**22**			1		
**23**			1	4	
**24**			1		
**25**			2		
**26**				6	
**27**				2	
**28**				1	
**29**				1	
**30**			1		
**31**					1

© 2017 The Authors. *Integrative Zoology* published by International Society of Zoological Sciences, Institute of Zoology/Chinese Academy of Sciences and John Wiley & Sons Australia, Ltd

Table S2 Number of *Curculio glandium* individuals bearing each haplotype in the five study populations

**Haplotype**	Maresme	Montseny	Olot	Prades	Ripoll
**1**	11	15	11	4	6
**2**	1				1
**3**			1		1
**4**	8	9	5	7	8
**5**	1	3		1	1
**6**		1			
**7**		1			
**8**			1		
**9**	1				
**10**	2				
**11**		1			
**12**		1			1
**13**	1				

Table S3 Mean ± SE density of host plants and the percentage of sound and infested seeds per location and host plant. Density of host plants was calculated as the mean of the nearest inventoried plots included in the Catalan Forest Inventory (Gracia *et al.* 2004).

	Q. pubescens			*Q. ilex*			*C. avellana*		
	Density (stems ha^{-1})	Sound (%)	Infested (%)	Density (stems ha^{-1})	Sound (%)	Infested (%)	Density (stems ha^{-1})	Sound (%)	Infested (%)
Ripoll	778 ± 64	25 ± 5	75 ± 9	-	-	-	341 ± 41	60 ± 4	40 ± 7
Olot	1021 ± 131	29 ± 11	71 ± 7	456 ± 33	45 ± 8	55 ± 11	441 ± 39	75 ± 8	25 ± 9
Montseny	678 ± 64	55 ± 9	41 ± 5	714 ± 81	60 ± 7	40 ± 5	456 ± 64	76 ± 11	24 ± 10
Maresme	512 ± 131	50 ± 8	50 ± 6	915 ± 44	59 ± 11	41 ± 6	501 ± 73	89 ± 12	11 ± 4
Prades	455± 96	64 ± 5	36 ± 7	875 ± 74	67 ± 6	33 ± 9	315 ± 84	76 ± 11	24 ± 8

© 2017 The Authors. *Integrative Zoology* published by International Society of Zoological Sciences, Institute of Zoology/Chinese Academy of Sciences and John Wiley & Sons Australia, Ltd
